# CCL28 induces mucosal homing of HIV-1-specific IgA-secreting plasma cells in mice immunized with HIV-1 Virus-like Particles

**DOI:** 10.1186/1742-4690-9-S2-P19

**Published:** 2012-09-13

**Authors:** V Rainone, G Dubois, V Temchura, K Uberla, M Nebuloni, E Lauri, D Trabattoni, F Veas, M Clerici

**Affiliations:** 1 University of Milan, Milan, Italy; 2 Faculty of Pharmacy, University of Montpellier, Montpellier, France; 3 Ruhr University Bochum, Bochum, Germany; 4 Don G. Gnocchi Foundation, Milan, Italy

## Background

MEC/CCL28 (CCL28) binds to CCR3 and CCR10 and recruits IgA-secreting plasma cells (IgA-ASCs) in the mucosal lamina propria. Virus-like Particles (VLPs) are a novel vaccine approach based on non-pathogenic particles that mimic the structure of virus particles with effective induction of both arms of the immune response. The suitability of CCL28 as an adjuvant for the elicitation of optimal mucosal and systemic immunity was assessed in mice immunized with HIV-1 VLPs.

## Methods

Balb/c mice were immunized intramuscularly with a prime-boost regime based on VLP containing gp160 from HIV-1 IIIB in the presence/absence of CCL28 and of the parental control CCL19. Flow citometry evaluation of CCR3 and CCR10 expression was performed on purified splenocytes. Th1 and Th2 cytokine production was performed on splenocytes and either colon, lungs or uterine cervix, whereas antigen-specific IgG and IgA antibodies were evaluated in sera and mucosal secretions by ELISA. Immune sera and mucosal secretions were tested for ex vivo neutralization activity against HIV-1 either subtype B or C strains. IgA-ASC recruitment at the mucosal level was verified with immune-histochemistry.

## Results

The following parameters were significantly augmented in VLP-CCL28 mice compared to control groups: the percentage and the surface density of CCR3 and CCR10 on CD19+ splenocytes; IFN-γ, IL-4 and IL-5 production in splenocytes and mucosal specimens; total IgA titers in sera and in mucosal secretions; antigen-specific IgG and IgA titers in sera and in mucosal secretions. Sera and mucosal secretions from VLP-CCL28 mice showed a significantly augmented neutralizing activity against homologous and heterologous viruses. IgA-ASCs were significantly increased in mucosal tissues of VLP-CCL28 mice.

## Conclusion

CCL28 used as an adjuvant has a robust immunomodulatory effect on potentially beneficial mucosal and systemic immune responses. These findings suggest that CCL28 could play a useful role in increasing the efficacy of preventive vaccines for mucosally transmitted viral infections.

